# Selection of reference genes for normalisation of real-time RT-PCR in brain-stem death injury in Ovis aries

**DOI:** 10.1186/1471-2199-10-72

**Published:** 2009-07-23

**Authors:** Margaret Passmore, Maria Nataatmadja, John F Fraser

**Affiliations:** 1 Department of Medicine, The Prince Charles Hospital, Chermside, Queensland, Australia; 2 Department of Intensive Care Medicine, The Prince Charles Hospital, Chermside, Queensland, Australia

## Abstract

**Background:**

Heart and lung transplantation is frequently the only therapeutic option for patients with end stage cardio respiratory disease. Organ donation post brain stem death (BSD) is a pre-requisite, yet BSD itself causes such severe damage that many organs offered for donation are unusable, with lung being the organ most affected by BSD. In Australia and New Zealand, less than 50% of lungs offered for donation post BSD are suitable for transplantation, as compared with over 90% of kidneys, resulting in patients dying for lack of suitable lungs. Our group has developed a novel 24 h sheep BSD model to mimic the physiological milieu of the typical human organ donor. Characterisation of the gene expression changes associated with BSD is critical and will assist in determining the aetiology of lung damage post BSD. Real-time PCR is a highly sensitive method involving multiple steps from extraction to processing RNA so the choice of housekeeping genes is important in obtaining reliable results. Little information however, is available on the expression stability of reference genes in the sheep pulmonary artery and lung. We aimed to establish a set of stably expressed reference genes for use as a standard for analysis of gene expression changes in BSD.

**Results:**

We evaluated the expression stability of 6 candidate normalisation genes (*ACTB, GAPDH, HGPRT, PGK1, PPIA *and *RPLP0*) using real time quantitative PCR. There was a wide range of Ct-values within each tissue for pulmonary artery (15–24) and lung (16–25) but the expression pattern for each gene was similar across the two tissues. After geNorm analysis, *ACTB *and *PPIA *were shown to be the most stably expressed in the pulmonary artery and *ACTB *and *PGK1 *in the lung tissue of BSD sheep.

**Conclusion:**

Accurate normalisation is critical in obtaining reliable and reproducible results in gene expression studies. This study demonstrates tissue associated variability in the selection of these normalisation genes in BSD sheep and underlines the importance of selecting the correct reference genes for both the animal model and tissue studied.

## Background

Lung transplantation represents the only prospect of improved survival and quality of life for patients with end stage pulmonary disease. Brain stem death (BSD) is a pre-requisite for the majority of heart and lung transplantation, yet this process adversely affects organ function, with lung being the most adversely affected. In Australia and New Zealand, less than 50% of lungs offered for donation post BSD are suitable for transplantation, as compared with over 90% of kidneys, resulting in patients dying for lack of suitable lungs [1]. We have been investigating the impact of BSD on pulmonary structure, remodelling and function to understand the process in the hope of ameliorating organ injury. Management of the BSD donor has been shown to positively impact both the number of organs which can be successfully transplanted and the function in these organs. Early organ dysfunction has a major impact on both short and long term survival as well as prolonged ICU and hospital stays, with the associated costs and risks of nosocomial infections in these immunocompromised patients. Hence, a clear understanding of the molecular changes associated with BSD may lead to further improvements of organs for transplantation. We have previously developed a 4 hour BSD model in the rat [2]. A clinically relevant model of BSD is an important key towards the understanding of the lung dysfunction post BSD, and we have subsequently developed a novel, clinically relevant 24 hour ovine model. The ovine models are treated in an animal ICU setting, with similar electrolyte management and hormonal resuscitation (methylprednisolone, tri-iodothyrosine (T3) and vasopressin) to mimic treatment given to human lung transplant donors prior to transplantation.

The haemodynamic changes in the systemic circulation in BSD are well described and are due to an initial catecholamine storm followed by relative hypotension, secondary to ischaemia of the sympathetic chain of the spinal cord [3]. We have previously described in the ovine model that pulmonary pressures post BSD rise by levels of 5 or more, as compared to 2–3 times in the systemic circulation [4]. The changes are more sustained and may therefore contribute to the changes in pulmonary microcirculation following BSD. Ongoing studies within our group are assessing changes in gene expression in both pulmonary artery and parenchymal tissue, as both may be affected by the same genes, and the differences in the localisation, organ structure and cell types needs to be taken into consideration. Similar gene expression levels in both organs will determine whether the pulmonary artery can be used as a valid representation of lung expression changes in the event of BSD. The determination of common stably expressed genes in both organs will enable reliable identification of gene expression changes in the donor lung prior to transplantation using excess pulmonary artery samples.

Gene expression in the organ donor is known to correlate to outcome post transplantation. In a recent study, gene expression of donor tissue prior to transplantation was correlated with severity of PGD and long term transplant outcome [5]. Gene expression studies using Real Time RT PCR are an integral part of understanding the impact BSD has on lung function. A clearer understanding of gene expression changes post BSD may lead to novel avenues of research to improve the lung function in both the donor and recipient. However, the donor population is inherently heterogeneous and the management varies substantially between centres. Use of an animal model allows for a more homogenous population and standardisation of management, minimising variation in assessing gene expression.

Accurate normalisation is critical in accounting for varying amounts of cDNA input and enzyme activity. As this is a novel ovine model, the first step in obtaining reliable data from this model is to standardise the housekeeping genes. The use of internal controls is the standard method for correcting for differences in input and enzyme activity and therefore the choice of internal standard is important in obtaining consistent and reliable results. Several studies have shown the importance of using multiple, stably expressed reference genes [6-10] and that changes in the housekeeping gene can lead to changes in the significance and expression of the target gene. We elected to use the geNorm program [11] which has been widely used by many researchers and has been statistically validated [12] to evaluate the expression of six candidate normalisation genes (*ACTB, GAPDH, HGPRT, PGK1, PPIA *and *RPLP0*) and their expression stability in pulmonary artery and lung.

The aim of this study was to develop a set of reference genes that can be used for normalisation of expression data in the pulmonary artery and lung of BSD sheep. The global aim is to develop a more complete understanding of the changes seen in the target gene population in the sheep post BSD.

## Results

**Figure 1:**
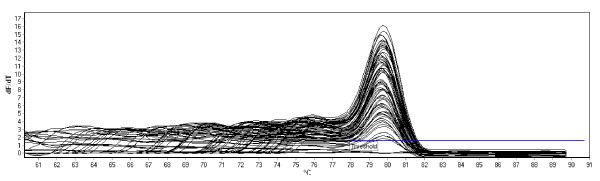
**Melt curve analysis**. Melt curve analysis of a representative gene ACTB showing the presence of one peak and a lack of non-specific fragments and primer dimers.

Primers were selected based on generation of the lowest cycle threshold (Ct-value – number of PCR cycles at which a significant increase in fluorescence is detected above background) and reaction specificity (Table 1). Analysis of melting temperatures showed only one peak and a lack of non-specific fragments and primer dimers. Melt curve analysis of a representative gene ACTB is shown in Figure 1.

**Table 1:** Primer information for candidate normalisation genes.

Gene	Forward primer (5' → 3')	Reverse primer (5' → 3')	Tm (°C)^a^	Amplicon size (bp)	PCR Efficiency^b^
ACTB	CCAAGGCCAACCGTGAGA	AGCCTGGATGGCCACGT	59	80	1.87
GAPDH	ATGCCTCCTGCACCACCA	AGTCCCTCCACGATGCCAA	60	76	1.88
HPRT	GCTGAGGATTTGGAGAAGGTGT	GGCCACCCATCTCCTTCAT	58	94	1.95
PGK1	ACTCCTTGCAGCCAGTTGCT	AGCACAAGCCTTCTCCACTTCT	59	101	2.03
PPIA	TCATTTGCACTGCCAAGACTG	TCATGCCCTCTTTCACTTTGC	59	72	1.78
RPLP0	CCAGGCTTTAGGCATCACCA	GGCGCCTACTTTGTCTCCTGT	60	94	1.96

^a^Theoretical melting temperature calculated with Primer Express software (Applied Biosystems).

^b^PCR efficiencies were determined using the formula 10^-1/slope^

We selected 6 commonly used reference genes *(ACTB, GAPDH, HGPRT, PGK1, PPIA *and *RPLP0*) of varying functional classes (for full gene information see Table 2). A dilution matrix of forward and reverse primers was performed in the range of 100 nM to 900 nM to determine the optimal concentration for each primer pair.

**Table 2:** Function of candidate normalisation genes.

Gene	Full gene name	Accession No.	Function	Primer conc (nM)	
				Forward primer	Reverse primer
ACTB	beta actin	U39357	Cytoskeletal structural protein	300	300
GAPDH	glyceraldehyde 3-phosphate dehydrogenase	AF030943	Carbohydrate metabolism	900	300
HPRT	hypoxanthine phosphoribosyltransferase	AF176419	Nucleoside, nucleotide and nucleic acid metabolism	300	300
PGK1	phosphoglycerate kinase 1	NM_001034299	Carbohydrate metabolism	100	100
PPIA	peptidylprolyl isomerase A	AY251270	Protein metabolism and modification	300	300
RPLP0	ribosomal protein, large, P0	NM_001012682	Protein metabolism and modification	900	900

**Figure 2:**
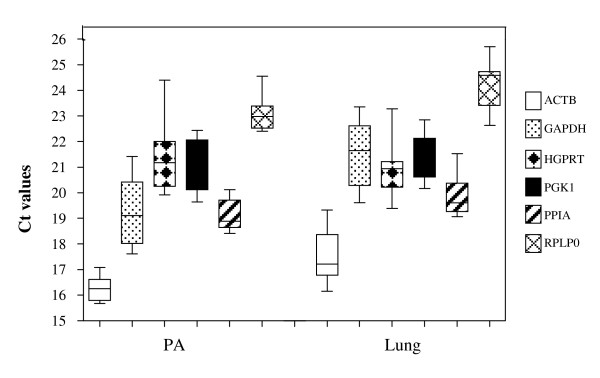
**Ct range for candidate normalisation genes in pulmonary artery and lung**. Values are given as real-time PCR cycle threshold number (Ct values) in pulmonary artery and lung of combined BSD and control samples. The coloured box represents 50% of the measurements for the gene. The thin, black line is the median and the whiskers represent the ranges for the data of 4 BSD and 4 controls.

The Ct-value of the candidate normalisation genes is shown in Figure 2. This shows a wide range of Ct-values within each tissue for pulmonary artery (15–24) and lung (16–25). However, the expression pattern for the majority of genes is remarkably similar across these two tissues, such that if a gene, like *ACTB*, is highly expressed in pulmonary artery (16.2), it is also highly expressed (17.5) in lung tissue.

**Figure 3:**
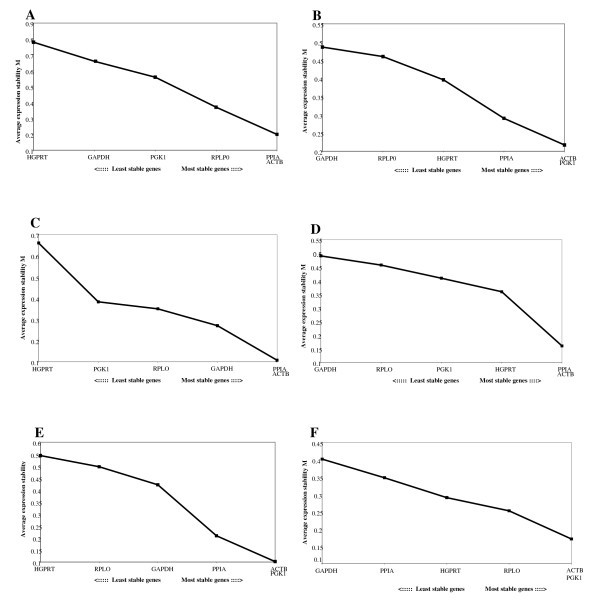
**Selection of candidate normalisation genes using geNorm analysis**. Stepwise exclusion of the least stable genes by calculating the average expression stability value M in combined BSD and control samples for pulmonary artery (A) and lung (B) and separate gene analysis for BSD pulmonary artery (C), control pulmonary artery (D), BSD lung (E) and control lung (F). The x axis indicates the ranking of the genes from least to most stable. The average expression stability value M is calculated for each gene with the highest M value indicating the least stable gene.

The geNorm program was used to identify the most stably expressed reference genes [11]. This program ranks candidate reference genes according to a stability value M. This value represents the mean pairwise variation between a candidate reference gene and all the other studied genes. The lowest M value indicates genes with the most stable expression. Stepwise elimination of successive genes showed that *PPIA *and *ACTB *were the most stably expressed genes in sheep pulmonary artery and *PGK1 *and *ACTB *were the most stably expressed genes in sheep lung (Figure 3). Further analysis was performed separating out the controls from the pulmonary artery and lung. When analysed separately the optimal reference genes for control and BSD pulmonary artery, and control and BSD lung were identical. This shows the high stability of these reference genes and that there is tissue-associated variability of gene expression in BSD, underlining the importance of choosing the most stable normalisation genes.

**Figure 4:**
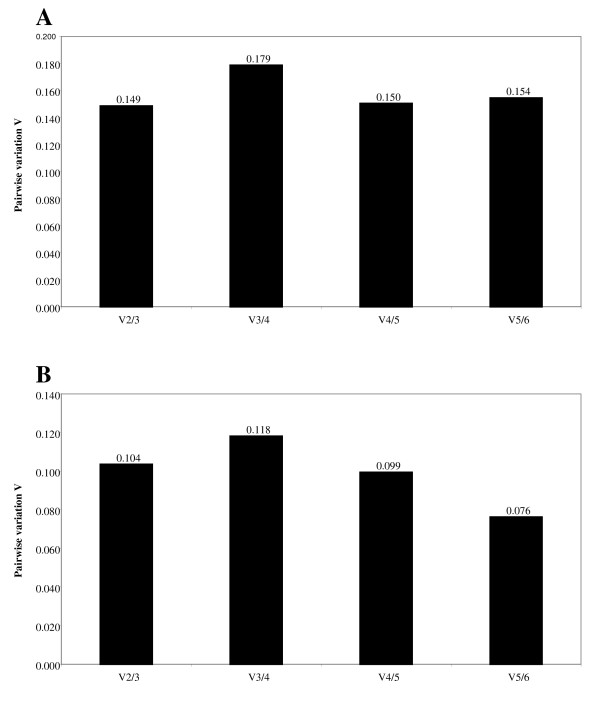
**Pairwise variation to determine the optimal number of normalisation genes**. The optimal number of genes was determined separately for pulmonary artery (A) and lung (B) in combined BSD and control samples. A large pairwise variation V indicates that the added gene has a significant effect and should be included in the normalisation factor calculation. Based on the pairwise variation for pulmonary artery and lung addition of a third reference gene does not improve normalisation in either tissue.

GeNorm was also used to determine the optimal number of reference genes for normalisation. The software calculates the normalisation factor (NF) from at least two genes at which the variable V defines the pairwise variation between sequential NFs. This means that V2/3 shows the variation of the NF of two genes in relation to three genes. We used a cut-off value of 0.15, below which the addition of an extra gene will not significantly improve normalisation [11]. Based on this assumption the addition of a third gene is not required for either pulmonary artery or lung (Figure 4) where the V2/3 values are 0.149 and 0.104 respectively. In fact, the addition of a third reference gene actually increases the pairwise variation to 0.179 for pulmonary artery and 0.118 for lung.

## Discussion

Real time RT PCR is a sensitive and accurate technique for measuring gene expression but it is important to correct for such factors as differences in sample input and enzyme efficiency. This can be achieved by normalising to a reference gene. Ideally a reference gene is ubiquitously expressed across all tissue types and under all experimental conditions. In sheep BSD however, little is known about the ideal genes to use for normalisation and many previous studies have only utilised a single reference gene in normalising gene expression data [13-15]. This is not ideal as variation in the housekeeping gene can lead to changes in the quantification of the gene of interest. The use of multiple reference genes is therefore important in obtaining accurate results, particularly in detecting small differences in gene expression. In order to achieve this we used the technique described by Vandesompele et al as a statistically validated technique which uses mean pairwise variation to determine the optimal number of reference genes.

We chose six commonly used reference genes from the literature. There was tissue-associated variability in the genes selected for normalisation, with *PPIA *and *ACTB *being the most stably expressed in pulmonary artery and *PGK1 *and *ACTB *the most stable in lung. When controls were analysed separately in the different tissue types the reference genes selected were identical showing a consistent level of expression for each reference gene in both control and BSD groups. Several studies have shown that housekeeping genes show variability within each tissue [16-18] emphasising that the stability of each housekeeping gene needs to be studied in each separate tissue and experimental set-up.

Interestingly *ACTB *is the most stably expressed gene in both pulmonary artery and lung. Traditionally this gene has been used in many studies to normalise gene expression data but recently has been shown to vary considerably depending on the cell type and tissue [18].

GeNorm analysis indicated that only two reference genes were needed for accurate normalisation. A cut-off value of 0.15 has been recommended [11] below which the inclusion of additional reference genes is not required. The addition of a third gene was shown to increase the variability in pulmonary artery and lung, therefore we used the geometric mean of the two most stably expressed genes.

The optimal number of subjects for such a study is unclear. The financial restraints and ethical considerations must be thought of simultaneously with the scientific merit of increasing the cohorts. Whilst a larger number in each study group may have been of interest, the high stability of the selected reference genes this study indicates that the chosen sample size is appropriate and at the very least serves as a good guide. This study lays the groundwork for a better understanding of the molecular changes occurring in BSD and ultimately in optimising donor management in the lung transplant population.

## Conclusion

This study identifies a new set of reference genes in pulmonary artery (*PPIA *and *ACTB*) and lung (*PGK1 *and *ACTB*) that can be used for normalisation of expression data in a 24 h BSD sheep model. It also demonstrates tissue-associated variability in the selection of these genes and emphasises the importance of selecting the most stably expressed genes across lung and pulmonary artery tissue for standard normalisation in gene expression studies.

## Methods

### Induction of BSD and sample collection

Eight sheep (4 controls; 4 BSD) were anaesthetised using Propofol (500–1200 mg/hr) and intubated with an endotracheal tube. All experimental procedures were conducted with the approval of the University of Queensland animal ethics committee (Reference PCH/389/06). Animals underwent placement of a urinary catheter, an arterial cannula followed by placement of a pulmonary arterial catheter. A size 10 tracheostomy (Portex™) was placed in the exposed trachea, the occiput cleared of adventitia and the bone plate exposed. An Intracranial Pressure "bolt" was introduced into a specifically designed rig to stabilise the device. A size 12 Foley catheter was introduced into a separate burr hole and then inflated with saline over a 30 minute period in the experimental animals. In the control animals the same procedure was followed except the catheter was not inflated. Animals were subsequently moved to specifically designed metabolic cages and sedation ceased at 12 hrs, whereupon hormone resuscitation was started with a 500 mg bolus of methylprednisolone, infusion of tri-iodothyrosine (T3) and vasopressin. At the end of the 24 hr period animals were euthanized while anaesthetised and pulmonary artery and lung specimens were immersed in RNA *later *(Ambion, CA, USA) overnight at 4°C and then kept at -80°C until RNA extraction.

### Isolation of RNA and cDNA synthesis

Total RNA was isolated using Trizol (Ambion, CA, USA) and samples purified with the RNeasy Mini Kit (Qiagen). All samples were DNase treated (Ambion, CA, USA) and subsequently analysed on an Agilent Bioanalyser (Agilent Technologies) to determine RNA concentration and quality [19]. Samples with a RNA integrity number (RIN) ≥ 6.5 were used. First strand cDNA was synthesized from 400 ng RNA using random primer and AMV Reverse Transcriptase (Roche, Basel, Switzerland).

### Real Time RT-PCR

Gene sequences were obtained from the NCBI database [20] and primers were designed using PrimerExpress (Applied Biosystems) and specificity checked using NCBI BLAST. Primers for HPRT, PGK1 and RPLP0 genes were based on areas of consensus between cow, human and sheep as only partial sequences were available for Ovis aries. The targets were evaluated for secondary structure formation using DNA calculator (Sigma) and primers were purchased from Invitrogen (Table 1).

Real time Quantitative PCR was performed using a Rotor-Gene 6000 real-time rotary analyzer (Corbett Research) with SYBR Green PCR Master Mix (Applied Biosystems). Reactions consisted of 5 μL SYBR Green Master Mix, 100 nM, 300 nM or 900 nM of forward and reverse primers and 2 μL of cDNA (equivalent to 20 ng) and nuclease free water to a final volume of 10 μL. The cycling conditions were as follows: cDNA was denatured at 95°C for 10 min, followed by 40 cycles of 95°C for 15 s and 60°C for 60 s (gain set at 10 for SYBR Green). Melt curve analysis was programmed at the end of the run, 60–90°C with increments rising by 0.5°C each step and a 5 s hold at each degree to determine reaction specificity and the absence of contamination, mispriming and primer dimer. Each PCR product had a single melt curve. A no-template and reverse transcription negative control was included for each primer set and PCR products were subsequently analyzed by agarose gel electrophoresis to check for correct products.

### Quantification and statistical analysis

Threshold cycle (Ct) values from the Rotor-Gene software version 1.7 (Corbett Research) were exported to Microsoft Excel for further analysis. All measurements were performed in triplicate for each gene and samples were quantified from standard curves, using serial dilutions of a cDNA pool of all PA and lung samples. This data was then analysed using geNorm 3.4 software to determine the most stable reference genes and the minimum number required to calculate a reliable normalisation factor using the geometric mean of multiple samples [11].

## Authors' contributions

MP performed all the experimental procedures and was the primary author of the manuscript. MN participated in the design of the study and critically revised the manuscript. JFF conceived the study design, performed all surgery, collected and processed samples and critically revised the manuscript. All authors read and approved the final manuscript.
